# Prostatic utricles without external genital anomalies in children: our experience, literature review, and pooling analysis

**DOI:** 10.1186/s12894-019-0450-z

**Published:** 2019-04-03

**Authors:** Bo Liu, Dawei He, Deying Zhang, Xing Liu, Tao Lin, Guanghui Wei

**Affiliations:** 1 Department of Urology, Children’s Hospital of Chongqing Medical University, No. 136, Zhongshan 2 RD, Yuzhong District, Chongqing, 400014 China; 2 Ministry of Education Key Laboratory of Child Development and Disorders, Chongqing Key Laboratory of Pediatrics, China International Science and Technology Cooperation base of Child Development and Critical Disorders, Chongqing Key Laboratory of Children Urogenital Development and Tissue Engineering, Chongqing, 400014 China

**Keywords:** Prostatic utricle cyst, External genital anomalies, Children

## Abstract

**Background:**

It has been recognized that the incidence of prostatic utricle in boys is increasing and is closely associated with diseases such as hypospadias. However, the clinical features of prostatic utricle with normal external genitalia have received little attention.

**Methods:**

Based on this, a series of 22 male children with prostatic utricles has been compiled by adding our 3 patients to 19 cases reported. All children enrolled had normal external genitalia. Clinical data of the case was reviewed.

**Results:**

Urinary tract infection, purulent urethral discharge and pyuria were the most common presenting chief complaint (41%), irritative lower urinary tract symptoms were present in 17% of cases, obstructive lower urinary tract symptoms were noted in 14%. Urinary retention has been reported in 18% and epididymitis has been reported in 14%. Relatively rare clinical symptoms are abdominal pain, hematuria, and hematospermia. A case of calculus formation and a case of neoplasia within the prostatic utricle has been reported. A cystic mass found by digital rectal examination is the most common presenting sign. A utricular lesion posterior to the bladder was revealed by imaging examination. Unilateral renal agenesis was associated in 32% of reports. Non-surgical approach was chosen in 3 cases, transrectal ultrasonography guided aspiration has been reported in 1 case. Endoscopic techniques were used in 3 cases. Open excision was used in 11 cases. The laparoscopic excision was chosen in 3 cases and Robot-assisted laparoscopy was reported in 1 case. Symptoms and signs disappeared after treatment in all children, and no recurrence occurred during follow-up.

**Conclusions:**

Prostate utricles without external genital anomalies are rarely reported in children, and are easily missed and misdiagnosed, often accompanied by recurrent urinary tract infections, lower urinary tract symptoms, epididymitis, dysuria and other symptoms. Imaging studies can confirm the diagnosis. Symptomatic and large utricles should be actively treated. The treatment program should be based on the age, clinical symptoms, and size and location of the utricle.

## Background

The prostatic utricle is an enlarged diverticulum in the posterior urethra, which result from incomplete degradation of the Müllerian ducts or decreased androgenic stimulation of the urogenital sinus [1]. Prostatic utricle is an uncommon congenital anomalies, with 1% incidence in autopsy findings and clinical prevalence of 5% in urologic patients [2]. Prostatic utricles are seen in young men commonly in the first and second decades and are associated with hypospadias, cryptorchidism, and pseudohermaphroditism/intersex disorders [3]. It is dived into grade 0-III according to the present feature based on voiding cystourethrogram (grade 0 - confined to the verumontanum; grade 1 - below the bladder neck; grade 2 - extend over the bladder neck; grade 3- opening distal to the external sphincter). The majority of prostatic utricles are asymptomatic. Symptomatic prostatic utricles may present with var. ious complaints including recurrent urinary tract infection, post-voiding dribbling, urethral discharge and epididymitis [4]. In particular, the presence of prostatic utricles in male patients with normal external genitalia has been reported but is exceedingly rare. Because these utricles are very rare, with few symptoms and no specific symptoms, a correct diagnosis is difficult to establish. Since no single series has been amassed for analysis, the clinical features, symptoms and signs of these patients, and how to deal with them have not been well characterized. Therefore, we combined the previously reported 19 cases with our own 3 cases for analysis to better clarify their clinical features and treatment management.

## Methods

All cases of prostatic utricles published in English from PubMed were reviewed, the closing date was June 30, 2018. All patients included in our study were children (age ≤ 18 years). Four cases were excluded due to the lack of required clinical data, and the remaining 19 cases (No. 4–22) and our own 3 cases (No. 1–3) constituted the subjects of this study [1, 4–20]. Clinical data of the case was reviewed with special attention to the patient’s clinical manifestations, diagnostic methods, therapeutic methods, pathological diagnosis and follow-up results.

## Results

### General information

The age of 22 patients with clinical symptoms ranged from 2 months to 18 years (mean age 5.8 years). Cases of the prostatic utricles have been reported in all races but are more common in white races (Table 1).

**Table 1:** Characteristics of prostatic utricles with normal external genitalia

Case No.- Pt. Age	Symptoms	Physical examination	Diagnostic procedure	Associated anomalies	Treatment performed
1 - 10 m	Recurrent purulent urethral discharge, recurrent UTI	Cystic rectal mass	Ultrasound, VCUG, MRI	None	Utricle catheterization and aspiration
2 - 15y	Acute urinary retention	Cystic rectal mass	Ultrasound, VCUG, CT	None	Utricle catheterization and aspiration
3 - 8 m	Recurring epididymitis	Cystic rectal mass	Ultrasound, VCUG, CT, MRI	None	Laparoscopic excision
4–4 1/2y	Difficulty voiding, low abdominal pain	Normal	Cystogram, cystoscope,	None	Retropubic, open operation
5 - 2 m	UTI	Normal	Ultrasound, VCUG, cystoscopy	Left renal agenesis, congenital obstructive posterior urethral membrane	Antimicrobial treatment
6 - 3y	UTI	Normal	IVP, cystoscopy, RUG	Left renal agenesis	Antimicrobial treatment
7 - 4y	UTI	Low abdominal mass	Ultrasound, urethroscopy,	None	Retrovesical, open operation
8 - 9y	Frequency, dribbling stream, daytime wetting, post-void fullness	Cystic rectal mass	VCUG	Vesicoureteral reflux	Posterior transsacral, open operation
9 - 10 m	Pyuria and fever	Cystic rectal mass, pus discharge on pressure	Ultrasound, cystoscopy	None	Transvesical, open operation
10 - 7y	Recurring epididymitis	Normal	Ultrasound, IVP, VCUG, RUG, cystoscope	Right renal agenesis	Transvesical, open operation
11 - 4y	Penile pain, low grade fever, urinary frequency, recurrent UTI	Mid swelling in the right testis, reproducible tenderness in the suprapubic area	Ultrasound, VCUG	None	Transvesical, open operation
12 - 16y	Intermittent, nonpainful, gross hematuria	Cystic rectal mass	CT, urethrocystoscopy	Right renal agenesis	Open operation
13 - 10y	Acute urinary retention	Distended urinary bladder	MRI, TRUS	None	TRUS guided aspiration
14 - 3y	Fever, lower abdominal pain, dysuria, acute urinary retention	Cystic rectal mass	MRI, cystoscopy	None	Laparoscopic excision
15 - 18 m	Recurrent UTI	Normal	RUG, cystoscope	Right renal agenesis	Laparoscopic excision
16 - 6 m	Retention and poor stream of urine	Palpable bladder	Ultrasound, VCUG	None	Posterior sagittal rectum retracting approach, open operation
17 - 6 m	Persistent purulent urethral discharge, recurrent UTI	Normal	Ultrasound, RUG, urethrocystoscopy	Right renal agenesis	Endoscopic utricle orifice incision
18 - 15y	Recurrent episodes of hematospermia	Normal	TRUS, MRI with an endorectal coil	None	Non-surgical approach
19 - 16y	Recurrent UTI, scrotal pain, low-grade fever, urinary frequency	Retrovesical mass	Ultrasound, MRI	None	Suprapubic extraperitoneal, open operation
20–1 1/2y	Recurrent epididymitis	Enlarged left-sided scrotal swelling	Ultrasound, CT, barium enema, RUG	None	Suprapubic extravesical and extraperitoneal, open operation
21 - 8y	Painful micturition, fever, progressive increasing abdominal girth	Cystic pelvic mass	CT	Left renal agenesis	laparotomy
22 - 6y	Urinary incontinence	Normal	Ultrasound, VCUG, MRI	None	Robot-assisted laparoscopy

CT, computed tomography; IVP, intravenous pyelogram; MRI, magnetic resonance imaging; RUG, retrograde urethrogram; TRUS, transcrectal ultrasound; UTI, urinary tract infection; VCUG, voiding cystourethrogram

### Clinical manifestation

Urinary tract infection, purulent urethral discharge and pyuria were present in 9 patients (41%). Irritative lower urinary tract symptoms (urinary frequency, urgency, dysuria and incontinence) were noted in 6 patients (17%). Obstructive lower urinary tract symptoms (Difficulty voiding, dribbling stream and poor stream of urine) were noted in 3 patients (14%). Urinary retention has been reported in 4 patients (18%) and epididymitis has been reported in 3 patients (14%). Relatively rare clinical symptoms are abdominal pain, hematuria, and hematospermia. A case of calculus formation and a case of neoplasia within the prostatic utricle has been reported. In 7 patients (32%), a digital rectal examination revealed a utricular mass above the prostate and posterior to the bladder. Less commonly reported signs include pelvic or abdominal mass and enlarged scrotal swelling.

### Associated congenital anomalies

All included cases have completely normal genitalia, unilateral renal agenesis was noted in 7 patients (32%). Less frequently patients have associated with congenital obstructive posterior urethral and vesicoureteral reflux.

### Diagnosis methods

**Fig. 1:**
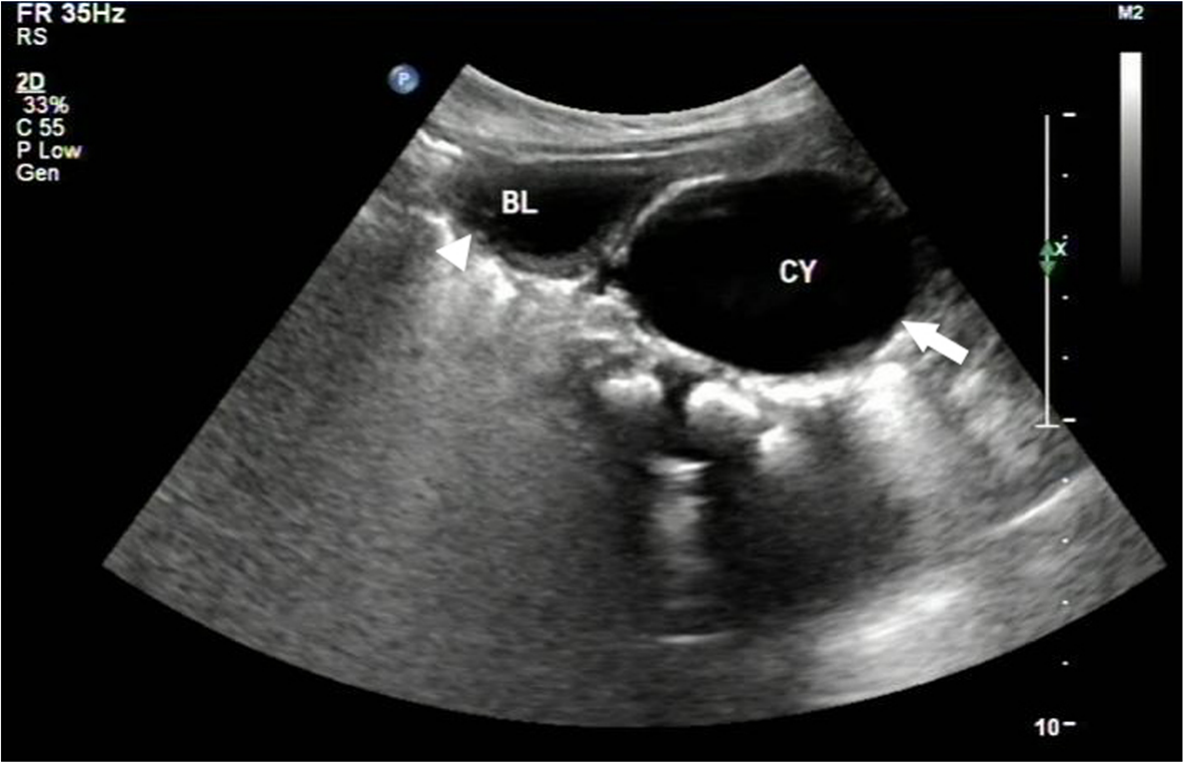
Transverse ultrasound of the pelvis shows a large utricle in the midline (white arrow), posterior to the urinary bladder (white arrowhead)

**Fig. 2:**
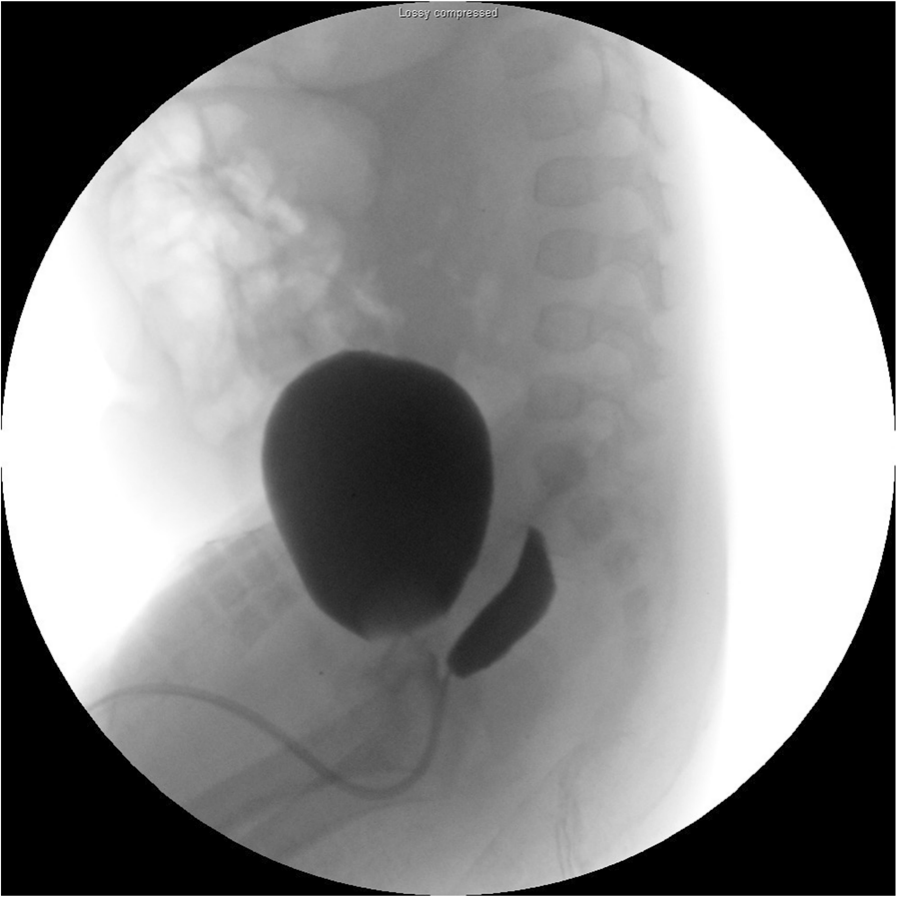
VCUG image depicts a prostatic utricle manifesting as a diverticulum that arises from the posterior wall of the urethra

**Fig. 3:**
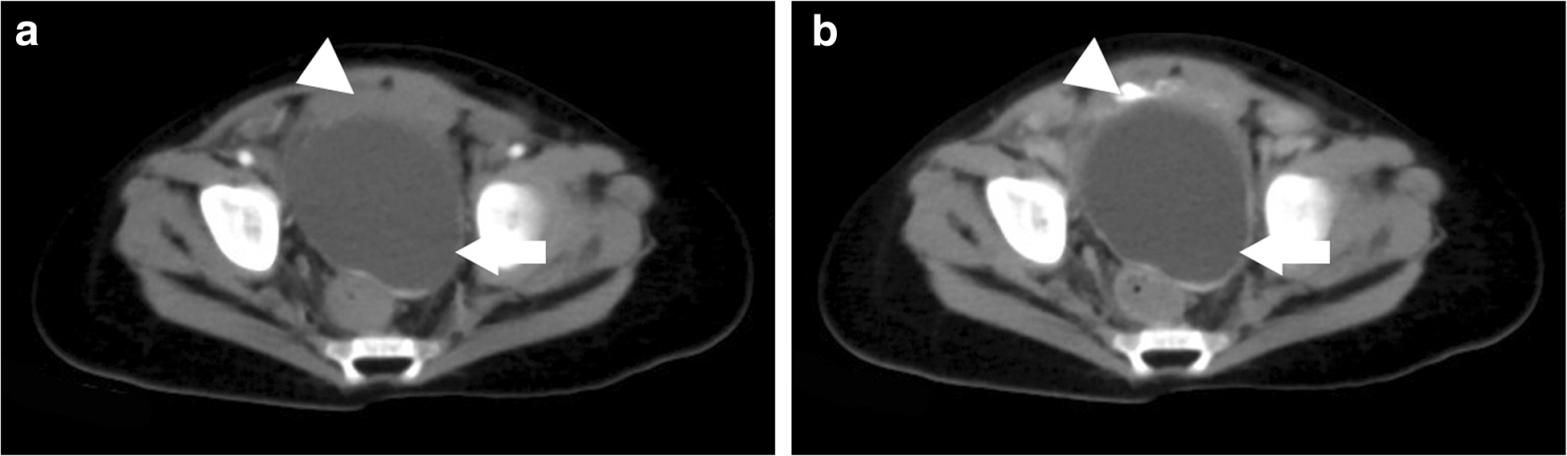
Contrast-enhanced computed tomography shows a large, utricular mass (white arrow) behind the urinary bladder (white arrowhead). **a** Enhanced CT shows a utricular retrovesical mass compressing the urinary bladder. **b** Delayed enhanced CT shows the bladder filled with contrast agent, a large utricular mass located behind and in close proximity to the urinary bladder

**Fig. 4:**
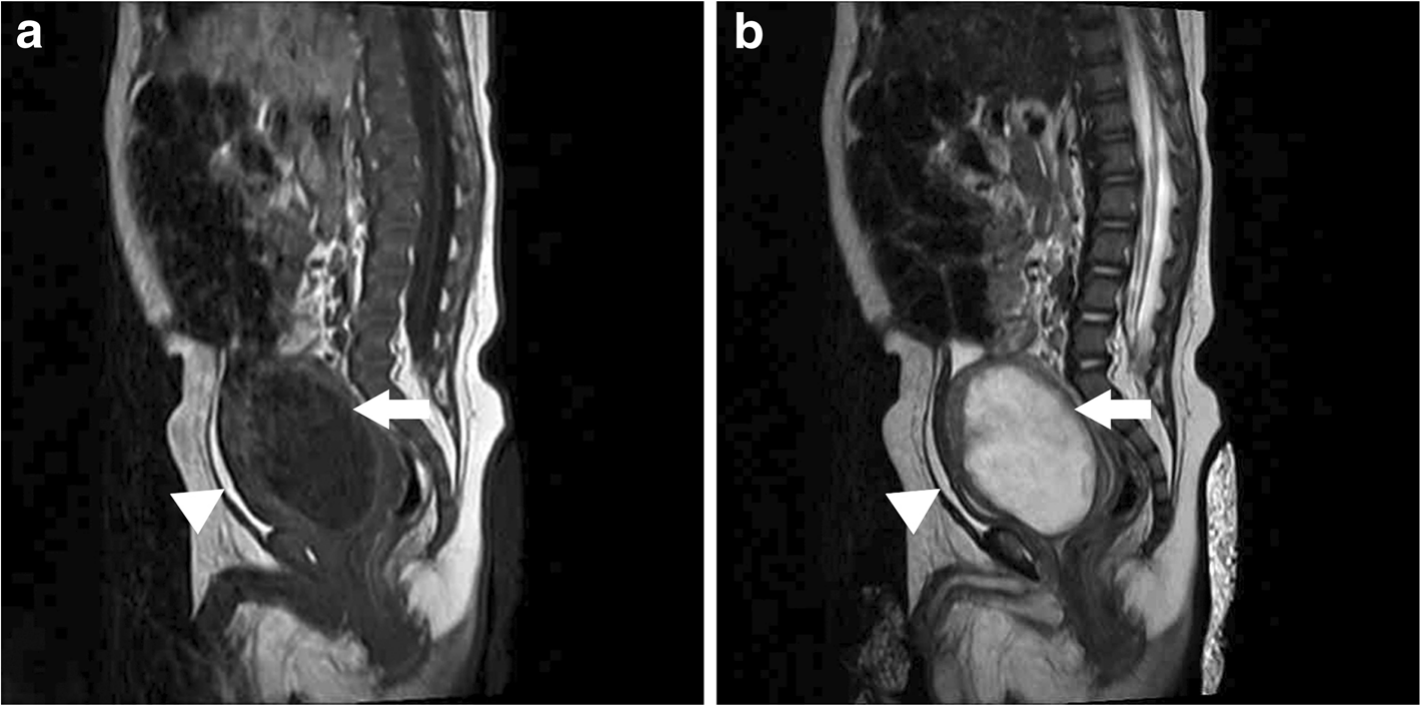
Magnetic resonance image shows a large prostatic utricle (white arrow) behind the urinary bladder (white arrowhead). **a** Sagittal T1 weighted MR image showing a hypointense lesion (white arrow) with thick and irregular wall posterior to urinary bladder (white arrowhead) and anterior to the rectum. **b** Sagittal T2 weighted MR image demonstrating a thick-wall, large utricular lesion behind the urinary bladder

Ultrasound was the most commonly used imaging method (Fig. 1), proving a correct diagnosis in 15 cases (68%). During voiding cystourethrogram (VCUG) or retrograde urethrogram (RUG) a utricular chamber filled from the posterior urethra in 13 cases (59%) (Fig. 2). Computerized tomography (CT) and magnetic resonance imaging (MRI) also made correct diagnosis in 11 cases (50%) (Figs. 3 and 4). Intravenous pyelogram (IVP) was used to auxiliary diagnosis of renal agenesis in 2 cases. Urethrocystoscopy revealed an orifice opening into a utricle in 10 cases.

### Treatments and outcomes

Non-surgical approach (including antimicrobial treatment) was chosen in 3 cases, transcrectal ultrasound (TRUS) guided aspiration has been reported in 1 case. Endoscopic techniques (utricle catheterization and aspiration and endoscopic utricle orifice incision) were used in 3 cases. Open excision (suprapubic extravesical, extraperitoneal, transvesical, retrovesical, retropubic, posterior transsacral, posterior sagittal rectum retracting approach) was used in 11 cases. The laparoscopic excision was chosen in 3 cases and Robot-assisted laparoscopy was reported in 1 case. No recurrence or repeated symptoms of the prostatic utricles were reported after any treatment.

### Pathology

In 15 cases of surgical excision, pathological findings were reported in 7 cases. Two of them were lined with squamous epithelium, one was lined with transitional epithelium and 1 was lined with flattened cuboidal epithelium. Two utricles were noted to have areas of squamous metaplasia. A rare clear cell adenocarcinoma was reported in a prostatic utricle.

## Discussion

The prostatic utricle is a rudimentary structure in the posterior urethra of males. After careful embryological investigation, the researchers believe that the cranial portion of the prostatic utricle is derived from the Müllerian ducts and the caudal segment has a mixed origin from the Müllerian ducts and Wolffian ducts and the urogenital sinus [13]. In males, the Müllerian ducts regress respond to Müllerian inhibiting factor (MIF) produced by the fetal testis, leaving them as a vestige. The urogenital sinus, which continues to form the distal third of the vagina in female, does not respond to MIF but instead masculinizes by closing off in response to testosterone in males [21]. Therefore, it is not surprising that the utricles were lined with squamous epithelium (Müllerian ducts), cuboidal epithelium and transitional epithelium (Wolffian ducts and urogenital sinus) in our reported histological examination. We also found that 32% of the cases show an association of unilateral renal agenesis, this may be due to the metanephric bud and renal blastema are in contiguity during the stage of prostatic utricle embryogenesis (7 to 8 weeks) [7].

The differential diagnosis need to be considered include Müllerian duct cyst, bladder diverticulum, urachal cyst, or a seminal vesicle cyst [22]. Among the diseases that need to be identified, Müllerian duct cysts are the most difficult to distinguish from embryology, clinical and imaging with the prostatic utricles because both of them are median intraprostatic cysts. Some researchers believe that the Müllerian duct cyst originates from the mesoderm, while the prostatic utricles originate from the endoderm [7]. Müllerian duct cysts are generally not connected to the prostatic urethra. They are round in shape and are often found in adults (20-40y) with normal external genitalia. Prostate utricles are tubular or vesicular in shape and most commonly seen in children (<20y) with hypospadias, cryptorchidism and gender dysplasia, usually communicating with the prostatic urethra [19]. Our patient is rare and unusual because all the features are in favor of a utricle that presented in the first or second period and communicated with the urethra but at the same time our patient had normal external genitalia. Two reasons are considered for the low number of reported cases. One is that the majority of prostatic utricles are asymptomatic, especially when small, thus symptomatic prostatic utricles are easily misdiagnosed or never diagnosed. Another is that symptoms of the prostatic utricle are varied and nonspecific, and outpatients are often treated symptomatically without further examination to determine the underlying disease. When large, the clinical presentation includes recurrent urinary tract infection, urethral discharge, post void urine dribbling, urinary retention, epididymitis, calculi formation and in rare cases malignant transformation [4].

The diagnosis is suspected when there is clinical manifestation and the mass is felt on the digital rectal examination. A pelvic ultrasound, a transrectal ultrasound or a perineal ultrasound can show cavity filled with fluid and its relationship with adjacent anatomical structures such as the prostate and urethra [23]. In VCUG or RUG, the utricles may have different sizes, showing an opacified cystic structure posterior to the prostatic urethra. However, it is worth noting that the identification of the prostate utricle by VCUG or RUG is often missed because the utricle is not fully filled, and the small prostate utricle is not easily detected by ultrasound. Fortunately, small prostate utricle (grade 0 and I) are often asymptomatic, and only need to be monitored without treatment. Enlarged prostatic utricles (grade II and III), which often requires surgical intervention, are easily detected by ultrasound, RUG and VCUG [24]. Using CT to detect these utricles is more accurate but it does not provide more help than ultrasound, RUG or VCUG. An MRI with an endorectal coil is particularly useful to delineate the utricle from the other pelvic structures due to their high resolution and multiplanar capability [25]. Certainly, the most useful investigation for a prostatic utricle is the urethrocystoscopy to identify the utricular orifice in the posterior urethra. Catheterization of the utricle with injection of contrast agent can be implemented to delineate the pouch more clearly [10]. Surgical treatment can also be performed with the aid of cystourethroscope.

Appropriate surgical procedures have been described to treat symptomatic and enlarged prostatic utricle. The first is endoscopic utricle orifice dilation, catheterization and aspiration, and resection of utricle roof. These methods have the advantage of less invasive, but also have a relatively high risk of recurrence [26]. We treated 2 of our patient with endoscopic utricle catheterization and aspiration. The reason for choosing this option was because the symptoms of the child were first seen, and also to avoid impotency and infertility caused by surgery. There was no recurrence by the adequate drainage of the wide mouthed diverticulum into the urethra. Surgical resection is recommended for utricles with recurrent symptoms and further neoplastic changes. Many open surgical approaches have been proposed to excise prostate utricle such as posterior pararectal [15]. Traditional open surgical approaches require high operational skills and may damage adjacent tissues [26]. Laparoscopic excision of prostatic utricle is suitable for surgeons who are skilled in advanced laparoscopic techniques. This technique reduces intrusion to the retrovesical space, provides a clear vision, and reduces the time required for recovery [26]. We have successfully applied laparoscopic techniques to a child with recurrent symptoms. Recently robot-assisted laparoscopy was considered as an advantageous technique for the treatment of prostatic utricle [20].

## Conclusions

The presence of a prostatic utricle in the absence of hypospadias, cryptorchidism or pseudohermaphroditism/intersex disorders is a rare finding. A combination of clinical signs and findings on physical examination guide diagnosis. Imaging test and urethrocystoscope can not only diagnose the prostatic utricle but also investigate its anatomical relationship. In general, small, asymptomatic, accidentally diagnosed prostatic utricles can be followed up without treatment. Complicated and large utricles need to be cured by surgery.

## Abbreviations

CT: Computerized tomography
MRI: Magnetic resonance imaging
RUG: Retrograde urethrogram
VCUG: Voiding cystourethrogram
